# Refugees in Sweden During the Covid-19 Pandemic—The Need for a New Perspective on Health and Integration

**DOI:** 10.3389/fpubh.2020.574334

**Published:** 2020-10-19

**Authors:** Mangrio Elisabeth, Paul-Satyaseela Maneesh, Strange Michael

**Affiliations:** ^1^Department of Care Science, Faculty of Health and Society, Malmö University, Malmö, Sweden; ^2^Malmö Institute for Studies of Migration, Diversity and Welfare (MIM), Malmö University, Malmö, Sweden; ^3^R&D Directorate, Acharya Institutes, Bangalore, India; ^4^Global Political Studies, Malmö University, Malmö, Sweden

**Keywords:** COVID-19, health care, health information, refugees, social situation

## Abstract

Refugees are already a vulnerable group in society and are in a stressful situation due to their often uncertain legal status in seeking asylum and integration in the new society after migration. Refugees are, in general, at greater risk of poor health outcomes when contracting Covid-19, exacerbated by poor living conditions and difficulties in accessing healthcare. The longer-term social consequences of the pandemic also disproportionately impact refugees, including social isolation, unemployment, and difficulties to obtain correct health information. The aim of this paper is to review the social and health consequences that Covid-19 has brought to the refugees residing in Sweden. This needs to be emphasized in order to mitigate against these likely consequences and improve the overall well-being among such a highly vulnerable group in society. As Covid-19 demonstrates, human health needs to be understood holistically, meaning that the vulnerability of any individuals, or even nations, is a vulnerability for the whole population requiring urgent action.

## Introduction

Refugees are already a vulnerable group in society and are in a stressful situation related to often lengthy and unpredictable asylum seeking processes as well as stress due to the challenges of integration in a new country (1, 2). This needs to be emphasized when the world is presently facing a pandemic and rapid spread of the novel severe acute respiratory syndrome coronavirus 2 (n-SARS-CoV-2) (3, 4).

As is now known, the spread of Covid-19 (coronavirus disease 2019) is reported to be from the Hubei province in China in late 2019, and in March-April 2020 the spread of this virus has reached the whole world leading to a pandemic. The virus has a higher degree of lethality than the endemic coronaviruses, though not to the level of SARS-CoV or MERS-CoV. The virus is now termed n-SARS-CoV-2 and causes the disease Covid-19 (5). Currently, there are worldwide > 2 million cases of Covid-19 resulting in >150,000 deaths (6).

Along with the rapid and intense spread worldwide of SARS-CoV-2, it is evident that more severe cases and deaths related to Covid-19 have underlying health conditions that contribute to the outcome (7). During a pandemic when there is no vaccine or proven treatment available, compounded with the uncertainty of immunity against the virus among the population, there is significant stress on the entire healthcare system that could potentially lead to higher mortalities from other normally treatable conditions. To protect the healthcare system and control the health impact of Covid-19, it is essential to identify and protect those groups particularly vulnerable to the pandemic. This paper focuses on refugees as a particularly vulnerable group, and uses Sweden as a case for the reason that the country has a high refugee population as well as having experienced significant exposure to the Covid-19 virus. The aim of this paper is to review the social and health consequences that Covid-19 has brought to the refugees residing in Sweden.

Refugees are a vulnerable group in society and it is reported that in Sweden this group has a higher proportion of overweight and obesity compared to the rest of the population, as well as higher smoking rates. This is a challenge since both overweight, obesity, and smoking have been seen as risk factors for Covid-19 (8, 9). Around 65% of the refugees in Sweden are either overweight or obese (10, 11) compare to 50% in the rest of the population (12). Among refugees in Sweden, around 35% are smokers (10, 11), which is higher than the rest of the population (12). Refugees also have challenges in accessing health care, and although they have the same rights to obtain care as the rest of the population, the proportion of unmet health care needs in this group is high (10, 11, 13). Unmet health care needs mean having been in need of health care during the last 3 months but not sought care (e.g., due to lack of trust in doctors, difficulties contacting medical services, etc.) (14). The proportion of unmet health care needs were between 30 and 70% in the different surveys (10, 11, 13).

A high proportion of refugees from Somalia and Eritrea have died in Sweden due to Covid-19 (15). These refugees typically reside currently in overcrowded socially deprived areas in Stockholm, Sweden, and often face linguistic barriers when accessing information necessary to adopting measures to mitigate their exposure to the pandemic (15). We also see that some refugee groups have low health-literacy, meaning knowledge about healthcare and personal well-being (14). These factors highlight the importance of better communicating information during a pandemic that needs to be sensitive to different populations and translated quickly for immigrant communities, as well as the broader health effects of poverty and social exclusion. There is also a potential for unemployment among the refugees due to their jobs being in small companies and restaurants; (14) this could lead the already vulnerable population being pushed into worsening poverty, since only around 30% of the refugees in Sweden were employed before the pandemic (10).

## Making Existing Problems Worse

Refugees often lack stable accommodation and employment, and have a greater risk of being criminalized and subject to punitive measures. Further on, along with creating significant stress for refugees, the demand to self-isolate within one's abode risks undermining measures to contain the virus unless regulators and policy enforcers are sensitive to the lack of stable accommodation available to refugees. Due to migration control measures, refugees regularly need to change address to avoid the risk of deportation, as well as often lacking access to reliable employment required to maintain access to stable housing. The effect is to force refugees into decreasingly livable conditions that, counterproductively, are likely to heighten their exposure to the Covid-19 virus and the life conditions identified as furthering its spread (16).

Refugees typically also have only limited access to health care and face administrative, legal, financial, or language barriers to accessing appropriate care in host countries (16). If there are no measures taken in order to improve conditions, the risk increases for an outbreak of Covid-19 among refugee groups (16). In general, worldwide refugees face a risk of increased societal stigmatization if they seek medical care and disclose any potential symptoms, fearing any potential diagnosis might worsen their already vulnerable status and civil freedoms (17). In Sweden, the heightened demand for big data surveillance in healthcare means that there are fewer channels through which refugees can safely seek help without fear of negative reprisals (18).

In considering Sweden, the approach has to a large extent continued to be an open society, which means that they have employed “soft measures” in dealing with the pandemic, mainly relying on recommendations and very few legislative controls (19). Strong recommendations related to hand hygiene, physical distancing, working from home when possible, staying home when sick and avoiding unnecessary traveling. Secondary schools and universities have been closed but preschool and primary schools open, since the children are not seen as the driver of the pandemic.

Due to the recommendation of physical distancing and less social gatherings, many businesses have either closed or scaled-down, especially within the hotel, restaurant, and travel sectors (20). This has, of course, an impact on employment in Sweden. The numbers of dismissals has increased and is above the numbers in one of Sweden's worst financial crises in living memory, that occurred 1990–1994 (20). This is a challenging situation especially for vulnerable groups such as refugees that already face challenges to find a job (10).

## Refugees as a Litmus Test

Vulnerable groups in society are historically made up of individuals who are the first to experience the negative effects of growing economic inequality and political instability. Refugees have acted as a litmus test of changing social norms, as decades of growing economic inequality and a declining welfare state were followed by new political parties which managed to articulate societal frustrations *via* an anti-migrant rhetoric that has seen them gain significant power across the globe (21).

As those often most excluded in society, refugees are a litmus test not only of less tolerant societal attitudes, but also of where the state begins to fail. Declined access to healthcare, for example, is felt first by refugees, since they are facing the greatest challenges to prioritize their own health needs and overcome the entry barriers (13). For example, in Sweden increasing demands on individuals—such as to use complicated technologies for booking healthcare appointments, or to take a greater role in deciding which healthcare service they should choose—can often function as barriers that prevent refugees accessing healthcare (22). In the case of Covid-19, where there is suddenly both an increased demand for healthcare services at the same time as new obstacles to seeking healthcare (e.g., cancellation of normal consultations, transfer of face-to-face meetings to telephony, and web-based meetings) there are few means through which refugees can obtain healthcare.

Whilst many of the negative effects of Covid-19 experienced by refugees are felt by the wider society of the host nation, due to the reasons outlined above it is often refugees that experience those problems first and to a greater extent. Refugees may come from countries where online activities are monitored closely by the police, there is a preference for exclusively face-to-face communication when dealing with private matters (23). Yet, the move over to digital-only solutions that has accelerated with Covid-19 means face-to-face communication is rarely an option with the risk that refugees choose to, instead, seek unreliable forms of health information and even self-medication.

Given this, refugees provide lessons for the broader society as to the effects of Covid-19 and where urgent action is required to mitigate the negative consequences.

## Refugees Post Covid-19

At this point in time, many possible worlds can be offered as credible future scenarios for a world post Covid-19 (24). But, what does the world post Covid-19 mean for this group? To the extent that refugees have often served as a political football to be kicked by different political parties seeking votes by transforming complex societal grievances into simple “us” vs. “them” narratives, the economic costs of Covid-19 may well only accelerate what has been a phenomena developed over several decades. Conversely, as it looks for now, so-called “populist” parties have seemed increasingly out-of-touch and irrelevant during the pandemic as the public have turned back to well-reasoned argumentation and research-driven journalism (25). The potential collapse of free movement within Europe, as well as a framing of Covid-19 as something “brought in” by migrants risks supporting anti-migrant voices, but such stories appear thin and false in many countries where there is widespread knowledge that, in most cases, the virus arrived via prosperous citizens returning from holidays or business trips, who then passed the virus onto the taxi drivers taking them home. That those taxi drivers were often refugees switches the populist claim that migrants are a threat, complicating what had been a once simple and powerful frame.

That refugees are asked now to be “good citizens” and respect the interests of the “community,” despite lacking citizsenship and being told they are not part of the community, adds a much greater nuanced perspective to how the public see such individuals (26). There are numerous counter examples, such as violent stigma against Chinese immigrants in Paris, but nevertheless the public are currently exposed to many more complicated accounts, as well as personal experiences, that undermine the kind of over-simplication that typically empowers anti-migrant politics. Wider exposure to the emotional trauma that comes with forced isolation, confinement, and loneliness mean many more now share the living conditions commonly endured by refugees.

Where refugees particularly experience the effects of border closures and generally restricted movements, rarely having access to jobs that might be performed from home, so too is this impact felt now by those industries reliant upon such transient workers. Agricultural sectors in Italy, for example, facing collapse due to a lack of such workers link the economy and food security of those affected countries to the ability of refugees to move freely and work. Again, simple narratives in which migrants are a “threat” have lost much of their, until recently, potent resonance. Whilst nations try to reassert their authority over the public good, we also see the importance of transborder trade flows, as well as human movement, in sustaining functioning societies. On an hourly basis individuals follow developments outside of their own borders, seeing lives lost or saved in India as indicative of their own life chances in the United Kingdom or Sweden, for example. On this basis, some have even suggested that Covid-19 might, by presenting the image of a “common humanity,” re-energize the movement for human rights (27).

## Discussion

This paper has highlighted the vulnerable situation that refugees overall are in and with special focus on refugees in Sweden, during the present Covid-19 pandemic. Refugees in Sweden are already facing challenges such as poor health, difficulties with employment, crowded living conditions and difficulties obtaining health care as well as understanding health care information (11, 13). This poses challenges during the current pandemic, since we have already in Sweden seen that migrants have died due to Covid-19 to a higher extent compared to the rest of the population. Precautions needs to be taken in order to avoid further deaths for this group in society.

It is therefore important to not exclude refugees from how we assess the societal costs of a health crisis such as the one we are now facing due to Covid-19. Refugees are already vulnerable, facing social and mental struggles and in addition struggling to enter the labor market (1, 10). These conditions have, for many, been worsened due to the Covid-19 pandemic. We also need to see the potential risk of domestic hardship within this group, due to mental and social struggles.

There is a need to find new ways to reach the refugees in Sweden with culturally sensitive and understandable health information, since we know that they already face challenges accessing health care (10, 11, 13, 28). Since we also know that refugees to a large extent are not trusting the health care workers, new ways of reaching the refugees could be through community leaders, representatives of migrant associations or other influencers in the community (19). We also need to ensure that refugees have the means to follow guidance, avoiding over-crowded and sub-standard accommodation that might foster the conditions conducive to the ongoing spread of Covid-19. This challenge on how to reach refugees during this pandemic is supported by Lancet Migration, that highlights the need to include refugees in the response to Covid-19 and to work toward decreasing the barriers that they face accessing prevention and health care during this pandemic (29).

While many countries that receive refugees encourage them to learn the official language of the country, in times of global emergencies such as a pandemic, it is very critical that the information is translated into the languages best understood by the refugees and disseminated among them as a priority to contain the spread of a pandemic (28). This is confirmed by Lancet Migration that highlights the need for accurate, linguistically and culturally appropriate public communication and information, alongside community mobilization (30). It should be reiterated that providing better information is insufficient if refugees are not economically supported to follow that information in their living conditions. As Covid-19 demonstrates, human health needs to be understood holistically, meaning that the vulnerability of any individuals, or even nations, is a vulnerability for the whole population requiring urgent action. Where that vulnerability is caused by state controls explicitly intended to be hostile to the welfare of refugees, in respect to healthcare and Covid-19 these policies hurt not only refugees but also the health security of the broader population by undermining efforts to control the current, and future, pandemics.

## Data Availability Statement

The original contributions presented in the study are included in the article/supplementary material, further inquiries can be directed to the corresponding author/s.

## Author Contributions

P-SM has given scientific contributions to the paper. All authors have read and accepted the final version of the paper.

## Conflict of Interest

The authors declare that the research was conducted in the absence of any commercial or financial relationships that could be construed as a potential conflict of interest.
